# PG545 sensitizes ovarian cancer cells to PARP inhibitors through modulation of RAD51-DEK interaction

**DOI:** 10.1038/s41388-023-02785-5

**Published:** 2023-08-07

**Authors:** Upasana Ray, Prabhu Thirusangu, Ling Jin, Yinan Xiao, Christopher L. Pathoulas, Julie Staub, Courtney L. Erskine, Keith Dredge, Edward Hammond, Matthew S. Block, Scott H. Kaufmann, Jamie N. Bakkum-Gamez, Viji Shridhar

**Affiliations:** 1 Department of Experimental Pathology and Medicine, Mayo Clinic, Rochester, MN USA; 2 Department of Obstetrics and Gynecology, Peking University Third Hospital, Beijing, China; 3 University of Connecticut Health Center-Medical School, Farmington, CT USA; 4 Department of Oncology, Mayo Clinic, Rochester, MN USA; 5 Zucero Therapeutics, South Melbourne, VIC Australia; 6 Department of Molecular Pharmacology and Experimental Therapeutics, Mayo Clinic, Rochester, MN USA; 7 Department of Obstetrics and Gynecology, Mayo Clinic, Rochester, MN USA

**Keywords:** Cancer, Autophagy

## Abstract

PG545 (Pixatimod) is a highly sulfated small molecule known for its ability to inhibit heparanase and disrupt signaling mediated by heparan-binding-growth factors (HB-GF). Previous studies indicated that PG545 inhibits growth factor-mediated signaling in ovarian cancer (OC) to enhance response to chemotherapy. Here we investigated the previously unidentified mechanisms by which PG545 induces DNA damage in OC cells and found that PG545 induces DNA single- and double-strand breaks, reduces RAD51 expression in an autophagy-dependent manner and inhibits homologous recombination repair (HRR). These changes accompanied the ability of PG545 to inhibit endocytosis of the heparan-sulfate proteoglycan interacting DNA repair protein, DEK, leading to DEK sequestration in the tumor microenvironment (TME) and loss of nuclear DEK needed for HRR. As a result, PG545 synergized with poly (ADP-ribose) polymerase inhibitors (PARPis) in OC cell lines in vitro and in 55% of primary cultures of patient-derived ascites samples ex vivo. Moreover, PG545/PARPi synergy was observed in OC cells exhibiting either de novo or acquired resistance to PARPi monotherapy. PG545 in combination with rucaparib also generated increased DNA damage, increased antitumor effects and increased survival of mice bearing HRR proficient OVCAR5 xenografts compared to monotherapy treatment in vivo. Synergistic antitumor activity of the PG545/rucaparib combination was likewise observed in an immunocompetent syngeneic ID8F3 OC model. Collectively, these results suggest that targeting DEK-HSPG interactions in the TME through the use of PG545 may be a novel method of inhibiting DNA repair and sensitizing cells to PARPis.

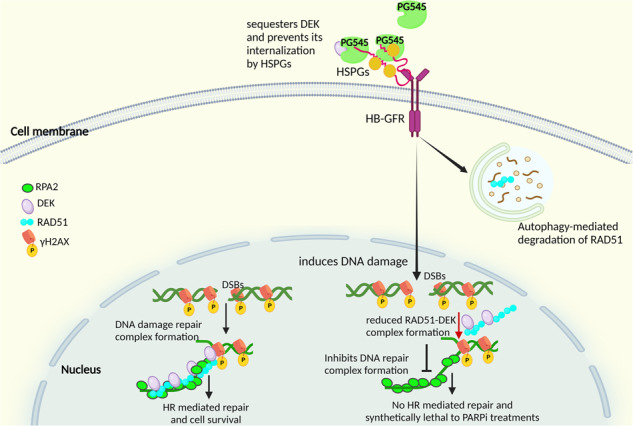

## Introduction

Recent advances in targeted therapies exploiting the common genetic alterations in HGSOC have led to FDA approval of PARP inhibitors (PARPis) as maintenance therapy to delay or prevent recurrence [1, 2]. PARPis disrupt multiple facets of DNA function, including repair of DNA single-strand breaks, resolution of stalled-replication forks, and joining of replicated fragments, generating double-strand breaks (DSBs) that require other pathways such as homologous recombination repair (HRR) and non-homologous end joining for repair [2]. Importantly, tumors with defective HRR, such as those that lack BRCA1 or BRCA2, are highly susceptible to PARPis because of limited capacity to repair these DSBs [2]. Although PARPi therapy extends median progression-free survival, mainly for OCs with HRR deficiency, several mechanisms of PARPi resistance have been identified in vitro and in the clinical setting. Thus, PARPi resistance is an emerging therapeutic challenge for OC patients; and there is a need for the development of therapies that can sensitize cancer cells to PARPis.

The small molecule therapeutic PG545 was designed with a core heparan-sulfate mimetic structure to target heparanase and heparin binding-growth factor (HB-GF) [3–5], a polypeptide that promotes angiogenesis, cancer metastasis, and chemotherapy resistance [3–9]. PG545 exhibited anti-cancer activity in a variety of several preclinical cancer models [5–12], including OC, and was well tolerated in a Phase 1a monotherapy trial in patients with advanced solid tumors (NCT02042781). Additional observations indicated that immunomodulatory effects also contribute to the anti-cancer effects of PG545 [4, 10, 13]. These findings, along with previous studies showing that inhibition of specific growth factor-mediated signaling pathways can increase PARPi sensitivity in HR-proficient OC cells, raised the question of whether PG545 can impact PARPi sensitivity.

In a separate line of investigation, heparan-sulfate proteoglycans (HSPGs) located at the cell surface have been shown to play vital roles in modulating the accessibility of HS-binding molecules such as HBGFs [14] as well as facilitating endocytosis of a diverse set of macromolecules [15]. Specifically, germane to the present study, HSPGs play a role in internalization of the oncoprotein DEK, a protein that is upregulated in a variety of cancers [16–18]. DEK in turn plays various physiological roles depending on its cellular localization. When localized to the nucleus, DEK has been implicated in chromatin remodeling [19, 20] as well as in HRR and the response to DNA replication stress [20, 21]. The secreted form of DEK, on the other hand, plays a direct role in inflammation as a chemoattractant for inflammatory cells [22, 23]. Importantly, secreted DEK can be internalized in a HSPG dependent manner [22].

Previous studies have demonstrated that DEK is overexpressed in OC and its knockdown decreases cell proliferation, induces DNA damage, and sensitizes cells to chemotherapy [24]. In the present study, which was designed to evaluate the effect of combining PG545 with PARPis, we report that PG545 impairs DEK uptake, leading to impaired HRR and enhanced PARPi sensitivity in vitro and in vivo in OC models.

## Results

### PG545 induces DNA damage and reduces HRR

**Fig. 1:**
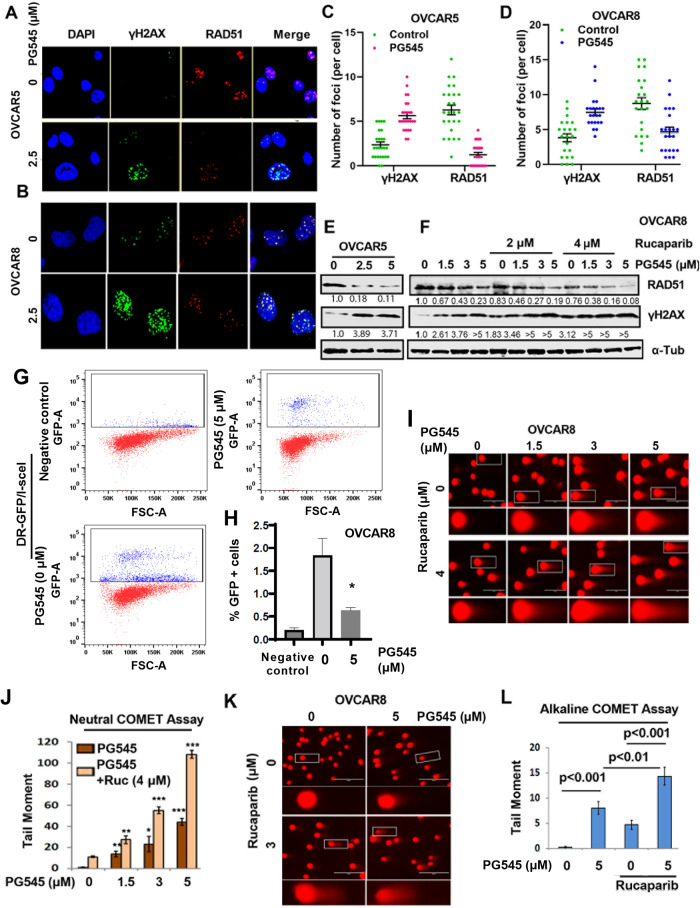
PG545 induces DNA damage in OC cells. **A** Representative immunofluorescence images of OVCAR5 and **B** OVCAR8 cells treated with 2.5 µM PG545 for 24 h. γH2AX (green), RAD51 (red) and nuclei were stained with DAPI (blue). **C**, **D** Quantification of 25 cells was performed in both the OVCAR5 and the OVACR8 cells respectively, and the number of foci per cell was scored and plotted as mean ± SEM (*p < 0.05 vs control). **E** Western blot analysis of dose dependent effect of PG545 treated for 24 h in OVCAR5 (**E**) and in OVCAR8 cells in presence of PG545 (+/−) 2 µM and 4 µM rucaparib (**F**) with αTubulin endogenous control. **G** Representative flow cytometric profiles of negative control (- the SceI plasmid), (–), and (+) PG545-treated cells after induction of the break within the DR-GFP that have successfully undergone HR directed gene conversion, forward scatter is shown on the x-axis and the intensity of GFP stain is shown on the y-axis. **H** Quantification of the proportion of GFP-positive cells after induction of I-SceI, *p < 0.05 vs. untreated cells. **I**, **J** Quantified Tail moment (represented as mean ± SD, *p < 0.05, **p < 0.01, ***p < 0.001 vs control) of the neutral COMET assay and **K**, **L** of the alkaline comet assay (represented as mean ± SD, ***p < 0.001 vs control, **p < 0.01 PG545 vs PG545 + rucaparib) in OVCAR8 cells treated with PG545 alone and in combination with rucaparib.

To better understand the role of PG545 in DNA damage response (DDR), we examined its impact on phospho-H2AX and RAD51 foci formation. Immunofluorescence (IF) showed that a 24-h PG545 treatment induced a marked increase in γH2AX and a concomitant decrease in the DDR protein RAD51 in OVCAR5 and OVCAR8 cells (Fig. 1A and C, B and D respectively). Immunoblot analysis cells after a 24-h PG545 treatment showed similar results in OVCAR5 cells (Fig. 1E). In OVCAR8 cells, on the other hand, the downregulation of RAD51 with PG545 alone was less extensive, but PG545 combined with rucaparib increased γH2AX and concomitantly decreased RAD51 levels more than either treatment alone (Fig. 1F). To determine the effect of PG545 on HRR, we used the DR-GFP reporter assay [25], which detects HR-mediated repair of the stably integrated HR substrate DR-GFP in OVCAR8 cells as restoration of full-length GFP that is quantified using flow cytometry. After induction of DNA DSBs with I-Sce1, significantly fewer GFP+ cells were found in PG545-treated cells compared to untreated cells, indicating inhibition of HR (Fig. 1G, H). Additionally, neutral and alkaline comet assays showed that combined treatment of PG545 with rucaparib induced more DNA damage in OVCAR8 cells than either monotherapy (Fig. 1I–L). Collectively these results indicate that PG545 impairs HRR and leads to greater levels of DNA damage.

### PG545 induces apoptosis and sensitizes OC cells to PARPi treatment

**Fig. 2:**
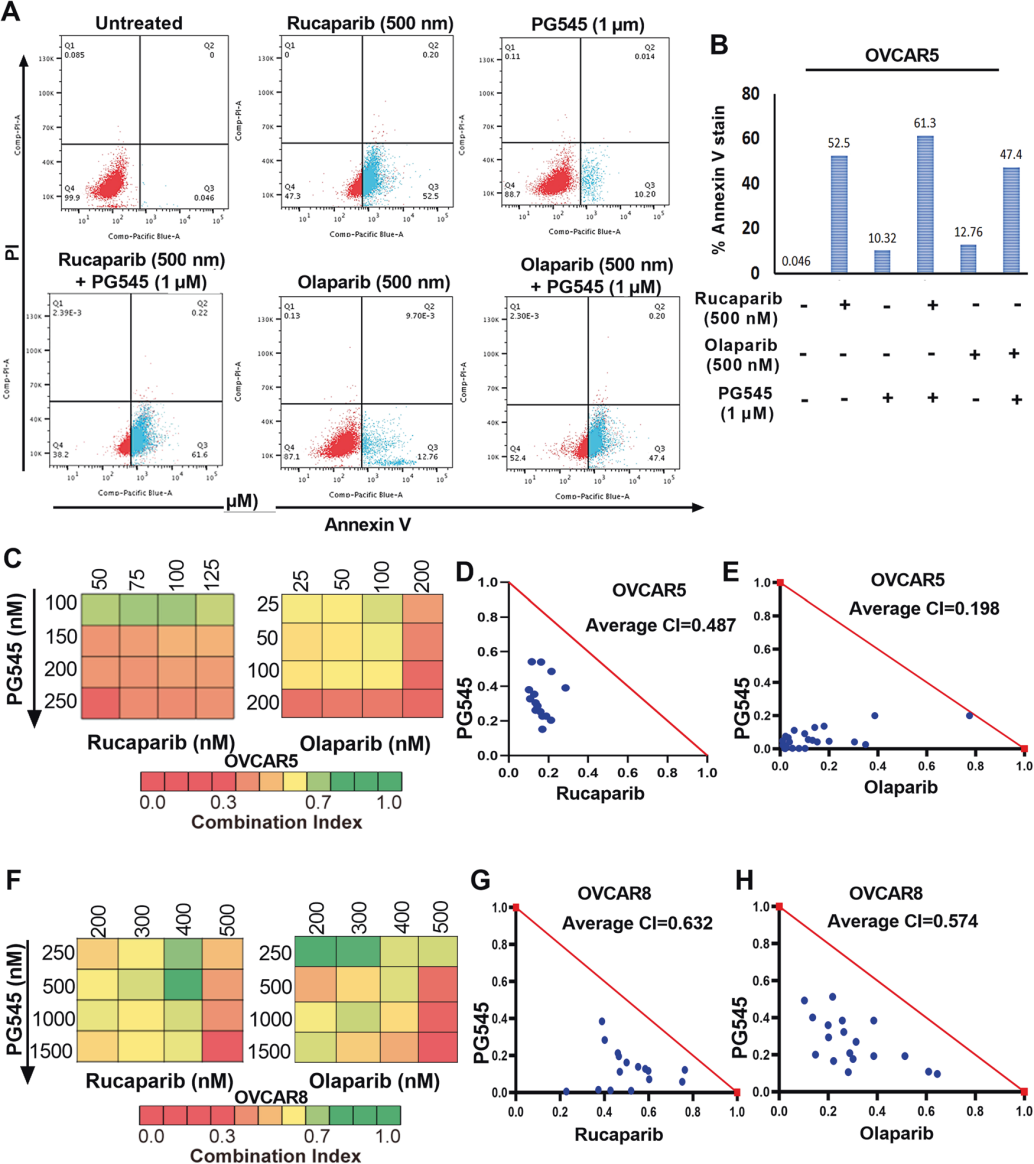
PG545 synergizes with rucaparib and olaparib to inhibit colony formation and promote apoptosis of OC cells. **A**, **B** Percent Annexin V+ cells from Flow cytometry analysis of Annexin V+ cells in OVCAR5 treated with 1 µM PG545 alone and in combination with either 500 nM rucaparib or olaparib, respectively. **C**, **F** Heat map from the results of Colony formation assays CFAs in OVCAR5 and OVCAR8 cells treated with the indicated concentrations of PG545 alone or in combination with rucaparib and olaparib showed synergy with combination index (CI) values <1. (**D**, **E** and **G**, **H,** respectively) The straight line is the additive isobole with synergistic dose combinations labeled below the isobole for rucaparib and olaparib in OVCAR5 and OVCAR8 cells respectively. An average CI of 1 indicates an additive effect, CI < 1 a synergistic effect, and CI > 1 an antagonistic effect.

Using clinically relevant concentrations, we observed increased staining with the apoptotic marker Annexin V in cells treated with PG545+rucaparib compared to either drug alone (Fig. 2A, B). Further analysis using colony formation assays (CFAs) indicated that the combination of PG545 with either rucaparib or olaparib was strongly synergistic in OVCAR5 (Fig. 2C–E) and OVCAR8 cells (Fig. 2F–H), with combination index (CI) values of 0.4–0.6 for PG545+rucaparib and 0.19–0.57 for PG545+olaparib, indicating that the HR-proficient OVCAR5 and OVCAR8 cells are sensitized to PARPi monotherapy by PG545.

In further studies, we performed CFAs on PEO1 cells, a *BRCA2*-mutant OC line, and PEO1/ABTr#3 cells, which exhibit acquired PARPi resistance (Table S2), including resistance to olaparib and rucaparib (Fig. S1A, B). Not only was the combination highly synergistic in the sensitive PEO1 parental cells (CI value 0.343) (Fig. S1C), but PG545 also sensitized the PEO1/ABTr#3 resistant cells to rucaparib, with a CI value of 0.527 (Fig. S1D). Collectively, these results indicate that PG545 synergizes with PARP inhibitors in both PARPi sensitive and PARPi resistant OC cells in vitro.

### PG545-induced autophagy sensitizes OC cells to PARPis

**Fig. 3:**
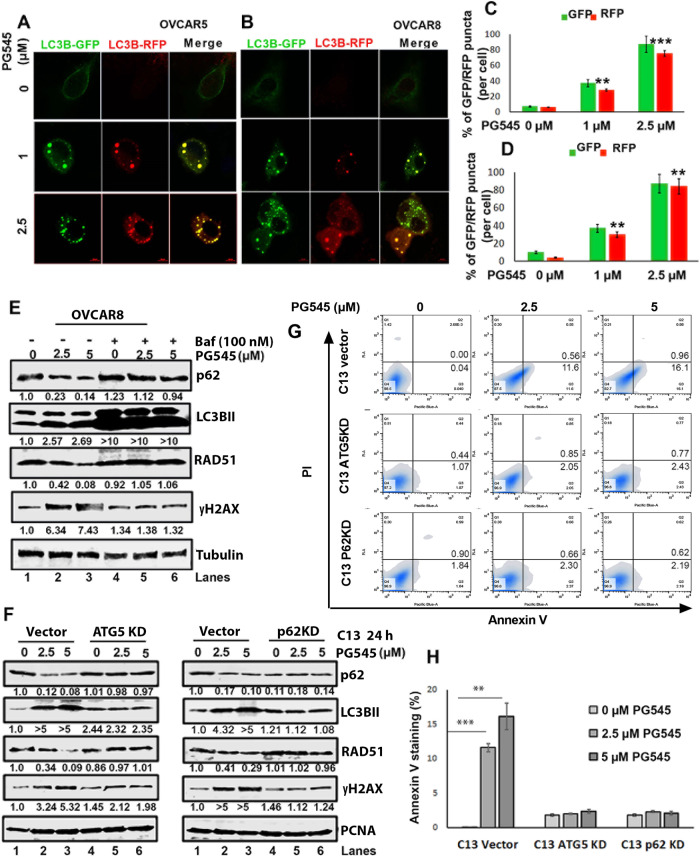
PG545 degrades RAD51 in an autophagy-dependent manner in OC cells. **A**, **B** After transient expression of a GFP-RFP-LC3B plasmid, autophagic flux (green GFP+ to yellow-red RFP+ GFP− puncta formation) was assessed in OVCAR5 and OVCAR8 cells treated with 1 μM and 2.5 µM PG545 for 24 h. **C**, **D** Calculated percent of GFP/RFP puncta per cell. **p < 0.01, ***p < 0.001 vs untreated. **E** Immunoblot analysis shows RAD51, γH2AX, P62 and LC3BII and control αTubulin levels in OVCAR8 cells treated with 2.5 and 5 µM PG545 with or without 100 nM BafA1 pretreatment. **F** RAD51, γH2AX, P62 and LC3BII levels were assessed in C13 cells transfected with empty vector or shRNA targeted to ATG5 or p62 and treated with 2.5 and 5 µM PG545 with PCNA as a loading control. Calculated fold changes (Image J software), normalized to endogenous control, are shown beneath each panel. **G**, **H** Flow cytometry analysis of Annexin V+ cells in the vector control vs ATG5 KD and p62 KD C13 cells treated with 2.5 and 5 µM PG545. Plot of percent Annexin V+ cells (represented as mean ± SD, **p < 0.01, ***p < 0.001 vs control).

Because PG545 can induce autophagy [8], we investigated whether PG545-induced autophagy may play a role in the PG545/PARPi synergy. PG545 increases autophagic flux in OVCAR5 and OVCAR8 cells as manifested by increased RFP+ puncta after GFP-RFP-LC3B transfection (Fig. 3A, C and B, D, respectively). Further, immunoblot analysis showed increased LC3BII levels and decreased p62/SQSTM1 in OVCAR8 cells upon PG545 treatment, indicating cells are undergoing autophagy (Fig. 3E). Under similar conditions, RAD51 is reduced (Fig. 3E), suggesting a potential role for autophagy in PG545-induced RAD51 downregulation. Additionally, treatment with BafA1, an inhibitor of late-stage autophagy, diminished PG545-induced RAD51 downregulation (Fig. 3E, lanes5–6). Moreover, the autophagy inhibitor chloroquine (CQ) diminished PG545-induced cell death in absence and presence of both rucaparib and niraparib (Fig. S2A–D) in OVCAR5 cells.

To confirm the importance of PG545-induced autophagy, we treated WT C13 cells and previously generated autophagy-deficient ATG5 knockdown (KD) cells (ref. [26] [Fig. 3C]) or p62/SQSTM1 KD cells (ref. [27] [Fig. 6A]) with PG545 (Fig. S2E). Immunoblotting showed PG545-induced downregulation of RAD51 in parental C13 cells but not in the ATG5 KD or p62/SQSTM1 KD cells (Fig. 3F). Additionally, PG545-induced apoptosis was attenuated in the autophagy-deficient cells (Fig. 3G, H). Moreover, the autophagy-deficient cells were more resistant to the PG545+PARPi combinations (Fig. S2F–H). Collectively, these results suggest that PG545-induced autophagy downregulates RAD51 levels, thus sensitizing the cells to PARPi treatment.

### Rad51 downregulation sensitizes cells to the PG545/PARPi combination

**Fig. 4:**
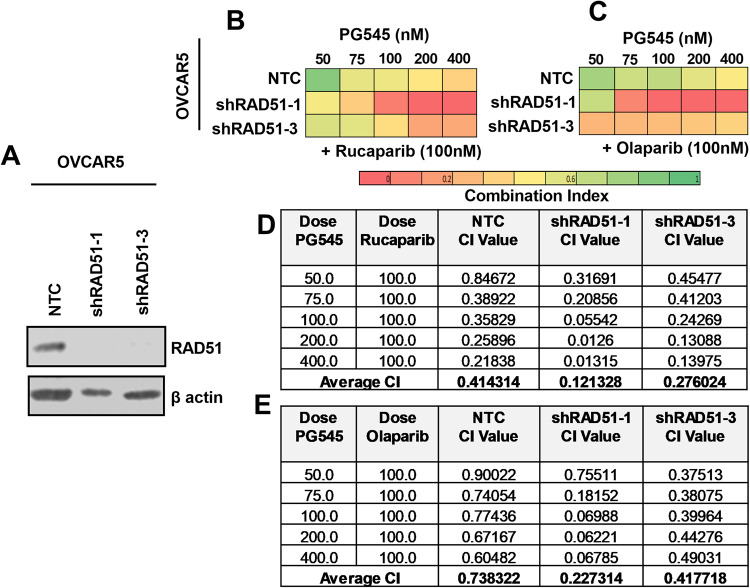
RAD51 knockdown cells are more sensitive to the PG545/PARPi combinations. **A** Immunoblot analysis of RAD51 expression in NTC control vs shRAD51 knockdown OVCAR5 cells with β-actin as loading control. **B**, **C** Heat map from the results of CFAs in OVCAR5 NTC (control) vs. OVCAR5 RAD51 KD cells treated with the indicated concentrations of PG545 alone or in combination with rucaparib and olaparib. **D**, **E** CI values across the treatment panel for rucaparib and PG545 and for olaparib and PG545 in NTC vs RAD51 KD OVCAR5 cells, respectively was shown. An average CI of 1 indicates an additive effect, CI < 1 a synergistic effect, and CI > 1 an antagonistic effect.

To further assess the role of RAD51 downregulation in the PG545/PARPi synergy, we knocked down RAD51 using two different shRNAs in OVCAR5 cells and using nontargeting control (NTC) shRNA as a control (Fig. 4A). CFAs indicated that the combination of PG545 with either PARPi was strongly synergistic even when RAD51 was knocked down (Fig. 4B, C), suggesting that PG545 might be sensitizing to PARPis rucaparib and olaparib (Fig. 4D, E respectively), by affecting other processes in addition to RAD51 expression. This prompted us to assess other potential mechanisms by which PG545 can affect cell survival.

### PG545 promotes extracellular DEK sequestration, preventing DEK-RAD51 nuclear co-localization

**Fig. 5:**
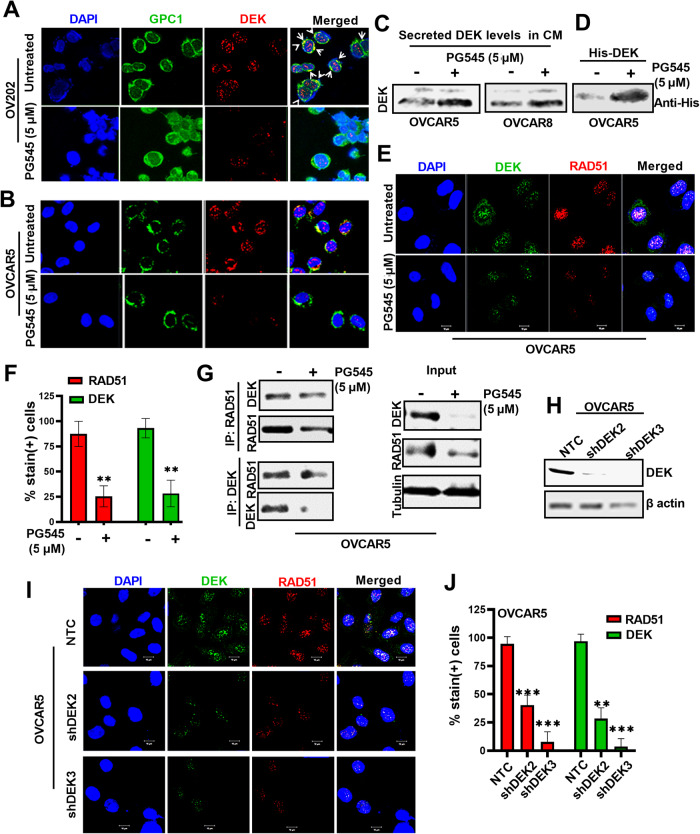
PG545 sequesters DEK in the microenvironment and attenuates nuclear DEK and RAD51 levels. Untreated and 5 µM PG545-treated IF images of **A** OV202 and **B** OVCAR5 against DEK (red) and GPC1 (green) and DAPI (blue) as nuclear stain. White arrows in merged panel **A** shows DEK bound to GPC1. **C** Immunoblot analysis of endogenous secreted DEK in conditioned media (CM) from OVCAR5 and OVCAR8 cells treated with 5 µM PG545. **D** Immunoblot analysis of transfected His_6_-DEK in CM from OVCAR5 cells treated with PG545 using anti-His_6_ antibody. **E** Representative confocal images showing DEK (green) and RAD51 (red) levels in OVCAR5 cells upon treatment with 5 µM PG545 (24 h). Nuclei stained with DAPI (blue). Scale bar: 10 µm. **F** Quantification of the % stain in images in (**E**). **G** PG545-treated OVCAR5 cell extracts were immunoprecipitated with anti-RAD51 and the co-precipitated DEK was detected by western blot analysis and vice versa. **H** Immunoblot analysis of DEK KD levels in shDEK2 and shDEK3 vs NTC control cells. Loading control, β-actin. **I** IF images showing DEK (green) and RAD51 (red) levels in NTC control and DEK KD OVCAR5 cells treated with 5 µM PG545 for 24 h. Nuclei stained with DAPI (blue). Scale bar: 10 µm. **J** Quantification of the IF images.

Because DEK is a secreted HS-binding protein, we hypothesized that PG545 might sequester DEK in the tumor microenvironment, thereby reducing its HSPG-mediated cellular uptake and nuclear localization [22, 23]. Treatment of OV202 cells that express high levels of the DEK-interacting HSPG glypican1 (GPC1, green in Fig. 5A) with 5 µM PG545 markedly reduced nuclear DEK (Fig. 5A, lower panel-Merge). Similar results were observed in untreated vs PG545-treated OVCAR5 cells (Fig. 5B). Instead, when either endogenous DEK or His6-tagged DEK produced from a transfected plasmid was followed, PG545 treatment was associated with accumulation of more secreted DEK in the medium (lower band, Fig. 5C, D).

To begin to examine the interaction between RAD51 and DEK, we performed IF and observed that these two proteins were colocalized in nuclei (Fig. 5E, F). In complementary studies, RAD51 immunoprecipitated DEK and vice versa (Fig. 5G), again suggesting association of these proteins. Further, stable KD of DEK in OVCAR5 cells (Fig. 5H) resulted in less DEK colocalized with RAD51 compared to control cells (Fig. 5I, J). Likewise, when localization of shRNA-resistant His_6_-tagged DEK was examined in DEK KD and NTC control cells, either PG545 or heparin reduced nuclear localization of His_6_-tagged DEK in the OVCAR5 shDEK cells (Fig. S3A), suggesting that PG545 as a HS mimetic sequesters DEK in the extracellular space, preventing its internalization. To further assess whether DEK has any direct role in promoting sensitivity to the PG545/PARPi combination, we performed cell viability assays in PEO1 NTC and DEK KD generated cells and observed that KD cells were more sensitive to the combination treatment (Fig. S3B–E). Together these results raise the possibility that PG545 attenuates DNA repair through two mechanisms, autophagy-mediated degradation of RAD51 and inhibition of DEK uptake required for recruitment of RAD51 at the damage site by DEK-RAD51 interaction.

### PG545 synergizes with rucaparib in a large subset of patient-derived ascites cells

**Fig. 6:**
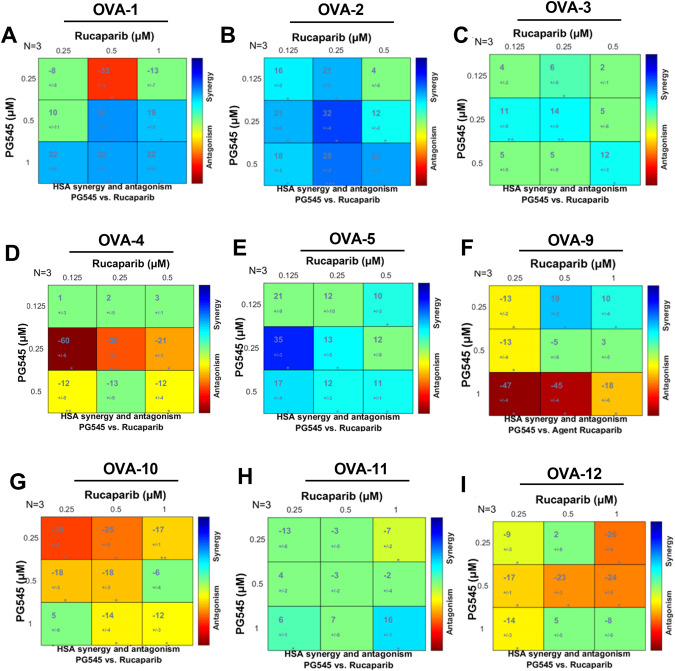
Effects of PG545 and rucaparib alone and in combination on patient-derived ascites cells in 3D culture. Dual-drug response assay of PG545 and rucaparib (number of biologic replicates *N* = 3) analyzed by the HSA synergy and antagonism matrix model using the Combenefit software. **A**–**I** The matrix format of synergy levels calculated according to the HSA synergy and antagonism model from the dual-drug experimental dose response in comparison to the reference dose-response surfaces respectively in patient-derived ascites samples OVA- 1, 2, 3, 4, 5, 9, 10, 11 and 12 respectively. The larger numeral in each box is the synergy score; negative values indicate antagonism. Boxes are colored green if the synergy score is not significant. The boxes colored according to the synergism/antagonism scale indicate results that are statistically significant by the one-sample *t*-test.

To assess the potential efficacy of the PG545/PARPi combination, we examined 9 patient-derived ascites samples. OC cells were enriched in epithelial cellular adhesion molecule (EpCAM) expression and exhibited little if any fibroblast activated protein (FAP) (Fig. S4A). The cells were grown as spheroids on extra-low attachment plates, exposed to PG545 ± PARPi for 72 h, and assayed for cell viability. After normalization to untreated samples, we plotted response surfaces indicating relative viability as a function of the two drug concentrations for each sample. Data were analyzed using the HSA additivity model implemented with combenefit software. The calculated CI values for individual drug combination for all tested ascites samples are shown in Table S4. This approach established that the highest synergistic effect of the two drugs occurred in OVA-1 and OVA-2 ascites cells (Figs. 6A, B and S5A, B; Table S4A, B). A moderate synergistic effect of the two drugs was also found against OVA-3, OVA-5 and OVA-11 ascites cells (Figs. 6C, E, H and S5C, E, H; Table S4C, E, H); while no synergism was found in OVA-4, OVA-9, OVA-10 and OVA-12 (Figs. 6D, F, G, I and S5D, F, G, I; Table S4D, F, G, I). Together these results indicate that PG545 sensitized ascites cells to PARPi treatment in ~55% of the samples tested.

### Synergistic antitumor effects of rucaparib and PG545 in vivo

**Fig. 7:**
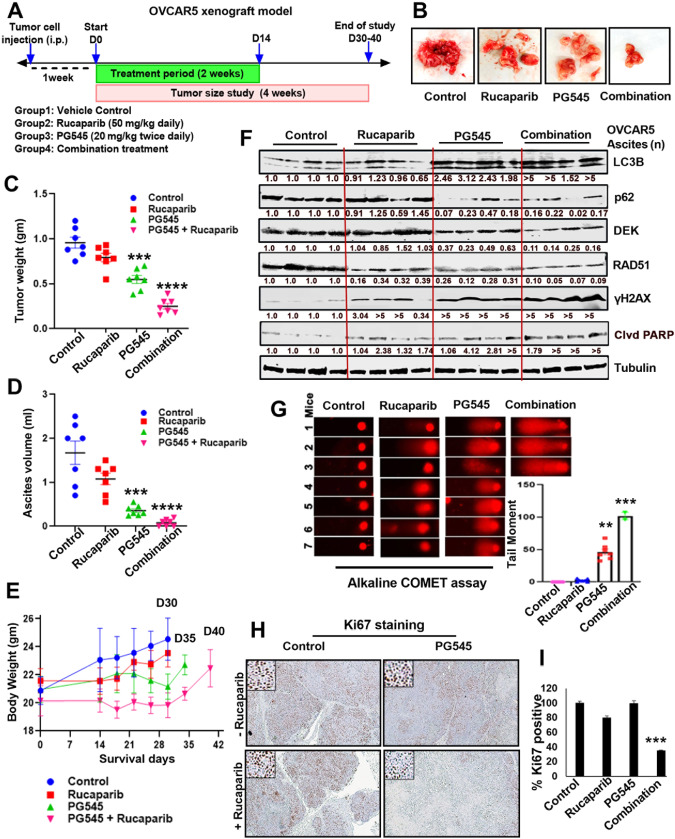
Rucaparib synergizes with PG545 to inhibit OVCAR5 xenografts in vivo. **A** Schematic representation of the in vivo study using the OVCAR5 OC xenograft. **B** Representative images of the excised tumors from one mouse from each of the four treatment groups. Graphical presentation of **C** the excised tumor weights, **D** ascites volume, and **E** body weight in the control vs treatment cohorts (*N* = 7, ***p < 0.001, ****p < 0.0001). **F** Western blot analysis of indicated markers from the ascites samples of the control and treated groups. Fold change was calculated using Image J software, normalized, and provided beneath the panel. **G** Alkaline COMET assay of the ascites samples from the control vs treated cohort of mice. Quantified tail moment is shown (represented as mean ± SD, **p < 0.01, ***p < 0.001 vs control). **H** Representative images of Ki67 staining in the tissue blocks of the four treatment groups. Scale bar: 300 µm. **I** Percent Ki67 positive was plotted. ***p < 0.001 vs control.

To assess whether the PG545/PARPi synergy occurs in vivo, the antitumor effect of PG545 alone and in combination with rucaparib was initially assessed using an orthotopic OVCAR5 OC xenograft model. Athymic nude mice injected intraperitoneally with OVCAR5 cells were randomized into four groups (n=7) and treated as shown in Fig. 7A. Representative excised tumors from a single animal in each group are shown (Fig. 7B). Comparative analysis of the tumor weight and ascites volume (Fig. 7C, D) showed that the combination led to reductions in tumor burden and ascites volume over the time. No significant body weight loss was observed among the groups (Fig. 7E). Western blot analyses of ascites-derived cells revealed decreased RAD51 and DEK along with increased γH2AX in the combination group compared to the monotherapy arms, as expected from in vitro studies; increased LC3BII and decreased p62 levels, indicating induced autophagy; and increased PARP cleavage, showing increased apoptosis (Fig. 7F). In addition, alkaline comet assays performed on ascites-derived cells showed increased DNA damage (tail moments) in the combination group compared to monotherapy groups, (Fig. 7G). Consistent with these data, IHC revealed decreased Ki67 staining of xenografts treated with the combination compared to monotherapy or control groups (Fig. 7H, I).

Because PG545 has a significant immunomodulatory effect, we also tested the efficacy of PG545 in an immunocompetent syngeneic ascites model developed using ID8F3 mouse OC cells. As shown in Figure S6A, mice were randomized into four groups and treated beginning 29 days after intraperitoneal inoculation. Comparison of the ascites volume in each group (Fig. S6B) showed that the combination produced a significant reduction in ascites volume compared to either monotherapy. Western blot analysis also indicated increased cleavage of caspase 3 and PARP1 along with a reduction in Ki67 levels in samples from the combination group compared to either monotherapy group (Fig. S6C).

Together, the results of the two in vivo models demonstrate that the PG545/rucaparib combination promotes extensive DNA damage that leads to enhanced antitumor activity in vivo.

## Discussion

In the present study we explored whether PG545, a highly sulfated small molecule that has activity in multiple tumor models and a tolerable safety profile in advanced cancer patients, can enhance PARPi sensitivity. Mechanistic studies using ATG5 and p62/SQSTMI KD cells showed that PG545 downregulated RAD51 expression in an autophagy-dependent manner, leading to increased accumulation of DNA damage. In addition, the present studies showed that PG545 diminished DEK accumulation from the TME and enhanced the cytotoxicity of PARPis in OC models in vitro and in vivo*.*

In preclinical studies and in the clinic, PARPi monotherapy is most active against HR-deficient cells. In contrast, PG545 was able to enhance PARPi-induced killing in both HR proficient (OVCAR5/8 and PEO1 ABTr#3) and HR-deficient lines (e.g., PEO1 and OVCAR5 shRAD51). In HR-proficient lines, the downregulation of RAD51 appears to contribute to the PG545-induced PARPi sensitization. Previous reports have shown that RAD51 is overexpressed in several cancers, including breast, pancreatic, non-small cell lung and prostate cancer expression, and is associated with lower overall survival in breast cancer [28], likely reflecting the contribution of RAD51 to HRR and consequent resistance to conventional cancer treatments [29]. Conversely, several studies have shown that RAD51 downregulation in HR-proficient cancer cells can lead to an HR-deficient phenotype and improve the effectiveness of current therapies [29], providing the rationale for current efforts to pharmacologically modulate RAD51 activity [30]. Accordingly, the effect of PG545 on RAD51 levels and PARPi sensitivity in HR-proficient cells is potentially interesting and merits further investigation.

Our further studies indicate that PG545 also diminishes the nuclear accumulation of the DEK oncoprotein [23, 24], which is secreted and then internalized by neighboring cells to promote HR repair [4, 6]. Importantly, it has been suggested that there might be a large therapeutic window for targeting DEK in cancer treatment, as differentiated epithelial cells are less sensitive than cancer cells to cytotoxic effects of DEK KD [24]. Even though DEK is overexpressed in a variety of other cancers [16–18], there are currently no therapeutic agents that target DEK. In this context, the present results showing that PG545 sequesters DEK in the extracellular space, thereby disrupting DEK internalization, nuclear trafficking and interaction with RAD51-containing complexes, might provide a means of targeting this oncoprotein in additional cancers beyond OC.

To further assess the PG545/PARPi combination, we examined it impact on tumor cells derived from 9 chemotherapy-naïve OC patients and tested in 3D culture. Our studies showed synergistic cytotoxic effects with the combination in 5 of 9 clinical isolates. The small number of samples analyzed constrained the statistical power, making it difficult to correlate the differences in response of ascites cells ex vivo to clinical characteristics or mutational status of the samples (Table S3). In these five samples, CI values tended to be lower with the PG545/rucaparib combination, which suggest synergistic possibilities in future pre-clinical studies. Importantly, in the 4 samples that failed to show synergy, rucaparib monotherapy failed to decrease cell viability, making it impossible for the Compusyn software to calculate CI values for the combination (indicated by NaN- Not a number in Table S4). Planned studies in additional samples are designed to identify the determinants of synergy to PG545/PARPi combinations with an eye toward prospectively identifying those tumors in which synergy occurs. Our data suggest that PG545 at readily achievable clinical concentrations can reverse PARPi resistance in vivo in two different OC models and in OC cells derived from >50% of patient ascites samples, suggesting that the present findings might have potential translational implications.

Our additional studies demonstrated activity of the PG545/rucaparib combination in an orthotopic OVCAR5 xenograft model. Given that PG545 has also been shown to possess immunomodulatory potential [10, 13], we also studied the effect of PG545 in an immunocompetent model using ID8F3 mouse OC cells and found that combination treatment with PG545/PARPi resulted in enhanced apoptosis and a significant reduction in ascites volume compared to either monotherapy treatment. Interestingly, extracellular DEK has been shown to be a chemotactic factor for T-cells and NK cells [23]. Thus, the sequestration of DEK in the TME by PG545 could be another mechanism by which PG545 promotes anti-cancer immunity. However, we did not explicitly investigate the effects of PG545 on immune infiltration in the present study, an effect that warrants further investigation.

While it is well established that PG545 disrupts HB-GF signaling, we found that PG545 also decreases RAD51 levels and sensitizes OC cells to PARPis. This is similar to recent preclinical studies reporting that inhibiting different tyrosine kinase receptors or their downstream signaling transducers can sensitize cancer cells to PARPis by reducing expression of BRCA1, BRCA2, RAD51, and other HR components, thereby preventing HR repair [31–33]. A phase 2 trial of cediranib+olaparib in relapsed platinum-sensitive OC showed increased survival compared with olaparib alone, with the survival benefit primarily in patients with wild-type *BRCA1/2* tumors [33]. While it is possible that disruption of HB-GF-signaling could similarly contribute to PG545-increased PARPi sensitivity in OC, our results suggest that the increased sensitivity also reflects additional effects on DNA repair as a result of the extracellular sequestration of DEK.

Collectively, the present data suggest that PG545 can impact HRR and reverse PARPi resistance in vitro and in vivo, suggesting that the present findings might provide the rationale to test PG545 as a single agent or in combination with PARPis in patients with OC.

## Materials and methods

### Reagents

PG545 was provided by Zucero Therapeutics (Brisbane, Australia). Olaparib and niraparib were purchased from LC Laboratories (Woburn, MA) and Chemietek (Indianapolis, IN), respectively. Rucaparib was kindly provided by Clovis Oncology (Boulder, CO). The concentrations used for all drugs in vitro and in vivo were therapeutically achievable [10, 34]. Other reagents and antibodies are listed in Table S1.

### Cell culture and drug sensitivity

Cell lines used in this study are listed in Table S2. To perform clonogenic assays, cells were seeded at 500 cells/well in 6-well plates, treated as indicated for up to 14 days until colonies became visible, stained with crystal violet, and quantified using ImageJ software [27].

Ascites samples from chemo-naive patients obtained at Mayo Clinic under an IRB-approved protocol (1288-03) or in collaboration with the University of Minnesota Cancer Center Tissue Procurement Facility with IRB approval (0702E01841) were cultured [35]. Briefly, ascites samples were centrifuged, and the cell pellet was trypsinized for 10 min. After centrifugation, the pellet was treated with 0.4% sterile ammonium chloride for 5 min to remove red blood cells and centrifuged again, washed twice with 1× PBS, and plated on low attachment plates using DMEM/F12 medium with 15% FBS. Clinical characteristics of patients providing ascites are provided in Table S3. After patient-derived ascites cells were seeded at 400 cells/well in 96-well low attachment plates and treated with the indicated drug concentrations for up to 3 days, cell viability was measured using CellTitre-Glo following the supplier’s protocol. It is the standard of care at our Institution to obtain germline genetic testing and/or somatic testing (e.g. Myriad myChoice CDx or FoundationOne CDx) for all newly diagnosed advanced stage and recurrent ovarian cancer patients. Based on the absence or presence of homologous recombination pathway gene mutations and homologous recombination deficiency scores, we determined the HRD status.

### Generation of stable knockdown clones

OVCAR5 cells were stably knocked down for DEK or RAD51 using specific targeted shRNA from Sigma Aldrich and following the supplier’s protocol.

DEK: sh2_Sequence:TGACTAAAGTACCAGATTATA, sh3_Sequence:AGGCACTGTGTCCTCATTAAA;

RAD51: sh1_Sequence:GCTAAGACTAACTCAAGATAA, sh3_Sequence:CCACAACCCATTTCACGGTTA

### Immunoprecipitation

Aliquots of OVCAR5 cell lysates containing equal amounts of protein were incubated with anti-DEK or anti-RAD51 separately for 24 h at 4 °C followed by protein A/G-agarose beads overnight. Samples were processed following the manufacturer’s protocol and probed for RAD51 or DEK as described in the Supplementary Material.

### Comet assay

Neutral and alkaline comet assays were performed following the indicated treatments using an established protocol [36]. Tail moments were quantified using ImageJ software.

### Autophagic flux assay

Cells were transiently transfected with GFP-RFP-LC3 plasmid and treated with the indicated PG545 concentrations for assessing the induction of autophagic flux as previously described [8]. Slides were then mounted and imaged using a Zeiss LSM 510 confocal microscope (Carl Zeiss, White Plains, NY).

### HR assay

OVCAR8 cells stably transfected with the HR substrate pDR-GFP [25] were transfected with pCβASceI plasmid, incubated for 24 h, treated with 5 µM PG545 for another 24 h, and analyzed for GFP fluorescence by flow cytometry.

### In vivo OC models

In vivo studies were performed in the OVCAR5 xenograft model using female athymic nude mice (nu/nu, 4–6 weeks old; Jackson Laboratory, ME) and the ID8F3 syngeneic xenograft model using female C57BL/6 mice [37]. Details of the treatment modalities and assessment are discussed in the Supplementary section. Animal experiments were carried out under the approved protocols and guidelines of the Mayo Clinic Animal Care and Use Committee.

### Immunohistochemistry

IHC studies for Ki67 staining were performed on formalin fixed paraffin-embedded sections as described [35] and imaged.

### Statistical analysis and synergy assessment

All experiments were performed in triplicates in each of 3 independent experiments unless indicated. The results were expressed as mean ± standard deviation. Statistical significance (*p < 0.05; **p < 0.01; ***p <0.001) was determined using Student’s *t*‐test unless otherwise noted. Synergy was assessed using Combenefit software [38], and combination index (CI) values were calculated using CompuSyn software applying a non-constant ratio approach [39] as described in the Supplementary Material. Notably, samples that failed to show synergy making it impossible for the Compusyn software to calculate CI values for the combination (indicated by NaN- Not a number in Table S4).

### Additional methods

Methods for immunofluorescence, immunoblotting, annexin V/PI staining, and in vivo OC models have been previously published and are found in the Supplementary material.

## Supplementary information

Supplementary information

## Data Availability

All data generated or analyzed for this study are included in this article.
