# Supplementary material for: PG545 sensitizes ovarian cancer cells to PARP inhibitors through modulation of RAD51-DEK interaction
Source: Oncogene. 2023 Aug 7;42(37):2725–36. doi: 10.1038/s41388-023-02785-5 (PMC10491494; doi:10.1038/s41388-023-02785-5)
Supplement: Supplementary file 1 — Supplementary information [file 41388_2023_2785_MOESM1_ESM.pdf]

PG545 sensitizes ovarian cancer cells to PARP inhibitors through modulation of RAD51-DEK interaction

Upasana Ray [1]*, Prabhu Thirusangu [1]*, Ling Jin [1]*, Yinan Xiao [1,2], Christopher L. Pathoulas [3], Julie Staub [1], Courtney L Erskine[4], Keith Dredge [5], Edward Hammond [5], Matthew S. Block[4], Scott H Kaufmann [4,6], Jamie N. Bakkum-Gamez [7] and Viji Shridhar [1]**

[1]Department of Experimental Pathology and Medicine, Mayo Clinic, Rochester, MN, USA

[2]Department of Obstetrics and Gynecology, Peking University Third Hospital, Beijing, China

[3]University of Connecticut Health Center-Medical School, Farmington, CT, USA

[4] Department of Oncology, Mayo Clinic, Rochester, MN, United States

[5] Zucero Therapeutics, South Melbourne, VIC, Australia

[6]Department of Molecular Pharmacology and Experimental Therapeutics, Mayo Clinic, Rochester, MN, USA.

[7]Department of Obstetrics and Gynecology, Mayo Clinic, Rochester, MN, USA

Table S1: List of Antibodies and Reagents

Primary Antibodies	Catalog No.	Company
DEK	#136222	Santa Cruz Biotechnology
PARP	#9542	Cell Signaling Technology
PCNA	#9857	Santa Cruz Biotechnology
RAD51	#133534	Abcam
Cleaved Caspase 3	#9661	Cell Signaling Technology
Histone H3	#517576	Santa Cruz Biotechnology
γ-H2AX	#26350	Abcam
LC3B	#3868	Cell Signaling Technology
α-Tubulin	#5286	Santa Cruz Biotechnology
β-Actin	#517582	Santa Cruz Biotechnology
p62/SQSTM1	#48402	Santa Cruz Biotechnology
GPC1	#365000	Santa Cruz Biotechnology
Ki-67	#9027	Cell Signaling Technology
GAPDH	#47724	Santa Cruz Biotechnology
Human epithelial specific antigen	CBL251	Chemicon International
Fibroblast activated protein	AF3715	R&D Systems, Inc

Reagents	Catalog No.	Company
3-(4,5-dimethylthiazol-2-yl)-2,5diphenyltetrazolium bromide (MTT)	M6494	ThermoFisher Scientific
fetal bovine serum (FBS)	#S181A	Biowest
Pacific Blue Annexin V	#640918	BioLegend
Propidium iodide	P1304MP	Thermo Fischer
Lipofectamine 3000	L3000015	Thermo Fisher Scientific
Antifade mounting medium with DAPI	H-1200-10	Vectashield, Burlingame, CA USA
DMEM (4.5 g/l glucose),<br>RPMI-1640<br>DMEM/F12	#11965118,<br>#11875093<br>#11330057	Thermo Fisher Scientific
100 µg/ml streptomycin and 100 U/ml penicillin	#15070063	Thermo Fisher Scientific
Bafilomycin A1	#54645	Cell Signaling Technology
CellTiter-Glo® 3D Cell Viability Assay	G9683	Promega

Table S2: Cell lines used in this study

Cell lines	Medium	Supplements	Source
OVCAR5	RPMI-1640	10% FBS and 1% Pen/Strep	American Type Culture Collection
OVCAR8	RPMI-1640	10% FBS and 1% Pen/Strep	Fox Chase Cancer Center
C13	RPMI-1640	10% FBS and 1% Pen/Strep	American Type Culture Collection
ATG KD C13[1]	RPMI-1640	10% FBS and 1% Pen/Strep	refer to reference 42, Figure 3C
p62/SQSTM1 KD C13[2]	RPMI-1640	10% FBS and 1% Pen/Strep	refer to reference 36, Figure 6A
OV202	DMEM (4.5 g/l glucose)	10% FBS and 1% Pen/Strep	Cheryl Conover, Mayo Clinic
PEO1	DMEM (4.5 g/l glucose)	10% FBS, 1% Pen/Strep, 10 μg/ml insulin, 1% nonessential amino acids	Fergus Couch, Mayo Clinic
PEO1/ABTr#3	DMEM (4.5 g/l glucose)	10% FBS, 1% Pen/Strep, 10 μg/ml insulin, 1% nonessential amino acids, 40 μM veliparib	Clonal line derived from PEO1 by continuous exposure to increasing veliparib concentrations up to 40 μM and subsequent cloning[3]

[1]As described in reference 42, this is a C13 cell line derivative with ATG5 knocked down.
[2]As described in reference 36, this is a C13 cell line derivative with p62/SQSTM1 knocked down.
[3]C. McGehee et al., ms. in preparation

All ovarian cancer cell lines used in this study, and their identity is routinely monitored to detect contamination and by short tandem repeat (STR) profiling through IDEXX BioResearch.

Table S3:

Patient samples	Category	Malignancy type	Histology	Stage	Substage	Grade	HR status

OVA-1	Malignant	Fallopian Tube	High Grade Serous	3	C	3	NA[1]
OVA-2	Malignant	Epithelial	High Grade Serous	3	C	3	HR+
OVA-3	Malignant	Primary Peritoneal	High Grade Serous	-	-	3	HR+
OVA-4	Malignant	Epithelial	High Grade Serous	3	C	3	HR+
OVA-5	Malignant	Epithelial	High Grade Serous	-	-	3	HR-
OVA-9	Malignant	Epithelial	High Grade Serous	4B	C	3	HR+
OVA-10	Malignant	Primary Peritoneal	High Grade Serous	High	NA	1	NA
OVA-11	Malignant	Primary Peritoneal	High Grade Serous	High	NA	3	NA
OVA-12	Malignant	Epithelial	High Grade Serous	3	C	-	NA

[1]Abbreviations: NA, not available; HR-, showing putative evidence of homologous recombination deficiency based on deleterious mutations or high HRD score; HR+, showing no evidence of homologous recombination deficiency.

Materials and methods

Immunofluorescence

Cells plated on chamber slides were treated as indicated in various figure legends for 24 hrs, fixed, permeabilized and stained with DEK, RAD51, GPC1, and γH2AX (1:100) antibodies overnight. The cells were washed, stained with fluorochrome-conjugated secondary antibody for 2-3 hrs, washed, stained with DAPI and visualized using a Zeiss-LSM510 confocal microscope.

Immunoblotting

Whole cell lysates were subjected to SDS-PAGE followed by western blotting [36] using antibodies listed in Table S1. Secondary staining was done with fluorophore-conjugated secondary antibodies (LICOR) and visualized using a LI-COR OdysseyFc Imaging System (Lincoln, Nebraska).

Annexin V/PI staining

Apoptosis was assessed as previously described [18] and analyzed on a FACSCalibur flow cytometer (Becton Dickinson, Franklin Lakes, NJ).

Synergy assessment

Synergy was assessed using Combenefit software (http://sourceforge.net/projects/combenefit/) [40], which calculates and shows the synergism-antagonism distributions and evaluates a range of metrics from the distributions. Drug assays of PG545 with rucaparib (N = 3) were analyzed by the HSA additivity model and colored when significant. Additionally, the Combenefit software was used to compare the experimental drug response surface to the reference surface and provide a percentage score to each cell in the matrix. Statistical significance was then provided by applying a one sample t test. Normalized isobolograms over a range of drug concentrations were generated,

and the combination index (CI) values were calculated using CompuSyn software (https://www.combosyn.com) applying a non-constant ratio approach, according to Chou and Talalay [41].

***In vivo* OC models**

Female athymic nude mice (nu/nu, 4–6 weeks old; Jackson Laboratory, ME) were injected intraperitoneally (i.p) with OVCAR5 cells (5×10^6 cells/mouse). Seven days following injection mice were randomized into 4 groups (n=7) for treatment as follows. Group 1: vehicle control; Group 2: Rucaparib (50 mg/kg daily by oral gavage); Group 3: PG545 (20 mg/kg twice weekly i.p); and Group 4: Rucaparib (50 mg/kg daily) + PG545 (20 mg/kg twice weekly). Mice were treated for 2 weeks and then followed for 2 weeks. We euthanized groups 1 and 2 on day 30 (due to 10% gain in body weight, humane endpoint) and group 3 on day 35, while group 4 mice were euthanized on day 40 because they displayed much lower tumor burden. The tumor weight and/or ascites volume were determined for all the cohorts. For the syngeneic xenograft model $5x10^6$ ID8F3 cells [39] were injected i.p. into 4 groups of female C57BL/6 mice (n=7) on Day 1. Beginning on Day 29, mice were treated with vehicle, olaparib alone (50 mg/kg by oral gavage daily), PG545 (20 mg/kg i.p. twice weekly) alone, or PG545 (20 mg/kg i.p. twice weekly)/olaparib (50 mg/kg oral gavage daily) for 14 days. A week after the end of treatment, the ascites was collected from each group. Animal experiments were carried out under the approved protocols and guidelines of the Mayo Clinic Animal Care and Use Committee.

A **OVA-1**

PG545 (nM)	Rucaparib (nM)	Combo CI
250.0	250.0	NaN
250.0	500.0	NaN
250.0	1000.0	NaN
500.0	250.0	0.83594
500.0	500.0	0.25767
500.0	1000.0	0.35582
1000.0	250.0	0.46587
1000.0	500.0	0.47573
1000.0	1000.0	0.49546

B **OVA-2**

PG545 (nM)	Rucaparib (nM)	Combo CI
125.0	125.0	0.20104
125.0	250.0	0.22514
125.0	500.0	0.81176
250.0	125.0	0.1438
250.0	250.0	0.11901
250.0	500.0	0.48061
500.0	250.0	0.21818
500.0	125.0	0.1533
500.0	500.0	0.29768

C **OVA-3**

PG545 (nM)	Rucaparib (nM)	Combo CI
125.0	125.0	0.63396
125.0	250.0	0.70359
125.0	500.0	0.85579
250.0	125.0	0.48749
250.0	250.0	0.52619
250.0	500.0	0.85313
500.0	250.0	0.70285
500.0	125.0	0.81936
500.0	500.0	0.76748

D **OVA-4**

PG545 (nM)	Rucaparib (nM)	Combo CI
125.0	125.0	1.20264
125.0	250.0	1.1223
125.0	500.0	1.27368
250.0	125.0	NaN
250.0	250.0	NaN
250.0	500.0	NaN
500.0	250.0	NaN
500.0	125.0	NaN
500.0	500.0	NaN

E **OVA-5**

PG545 (nM)	Rucaparib (nM)	Combo CI
125.0	125.0	0.45717
125.0	250.0	0.42413
125.0	500.0	0.6378
250.0	125.0	0.53039
250.0	250.0	0.6815
250.0	500.0	0.7789
500.0	250.0	1.41114
500.0	125.0	1.29965
500.0	500.0	1.45767

F **OVA-9**

PG545 (nM)	Rucaparib (nM)	Combo CI
250.0	250.0	NaN
250.0	500.0	NaN
250.0	1000.0	NaN
500.0	250.0	NaN
500.0	500.0	NaN
500.0	1000.0	NaN
1000.0	250.0	NaN
1000.0	500.0	NaN
1000.0	1000.0	NaN

G **OVA-10**

PG545 (nM)	Rucaparib (nM)	Combo CI
250.0	250.0	NaN
250.0	500.0	NaN
250.0	1000.0	NaN
500.0	250.0	NaN
500.0	500.0	NaN
500.0	1000.0	NaN
1000.0	250.0	NaN
1000.0	500.0	NaN
1000.0	1000.0	NaN

H **OVA-11**

PG545 (nM)	Rucaparib (nM)	Combo CI
250.0	250.0	4.68005
250.0	500.0	0.77986
250.0	1000.0	1.21542
500.0	250.0	0.50925
500.0	500.0	0.829
500.0	1000.0	1.02845
1000.0	250.0	0.45259
1000.0	500.0	0.56132
1000.0	1000.0	0.54462

I **OVA-12**

PG545 (nM)	Rucaparib (nM)	Combo CI
250.0	250.0	NaN
250.0	500.0	NaN
250.0	1000.0	NaN
500.0	250.0	NaN
500.0	500.0	NaN
500.0	1000.0	NaN
1000.0	250.0	NaN
1000.0	500.0	NaN
1000.0	1000.0	NaN

Table S4: Synergy studies showing CI values with PG545 and rucaparib in combination using patient-derived ascites cells. Panels A-I showed the CI values for the different dose combinations in ascites samples OVA- 1, 2, 3, 4, 5, 9, 10, 11 and 12. CI value of 1 indicates an additive effect, CI < 1 a synergistic effect, and CI > 1 an antagonistic effect.

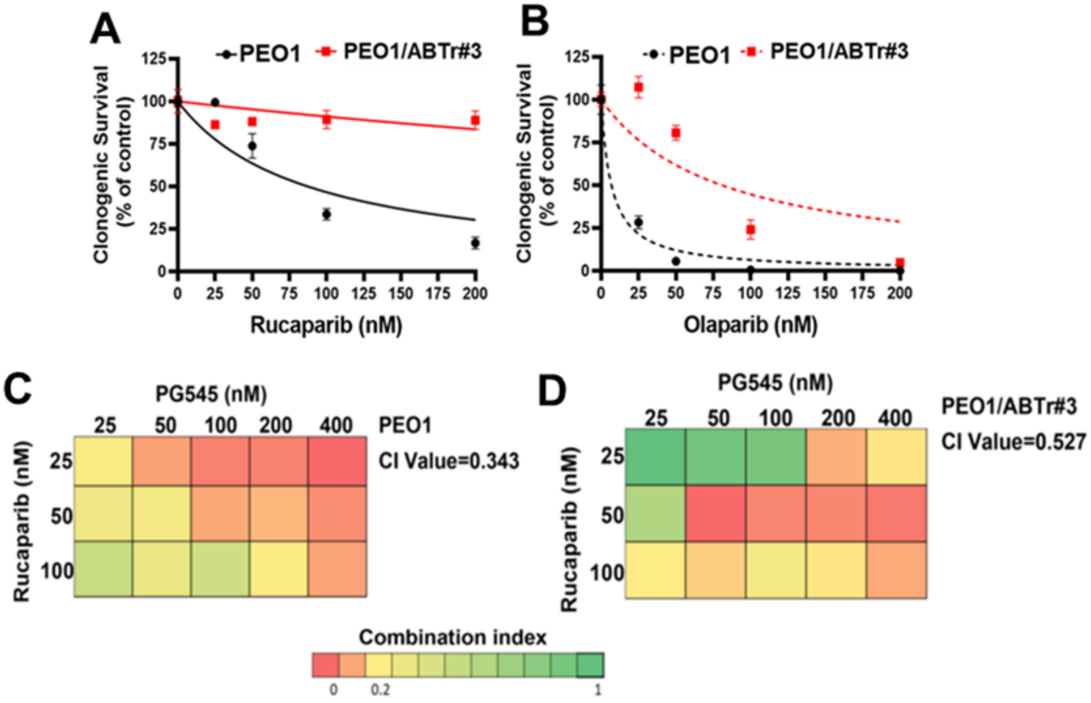

Supplementary S1: PG545 treatment sensitizes the PARPi sensitive and PARPi resistant cells to the combination treatment. (A-B) CFAs were performed using PEO1 and PEO1/ABTr#3 cells treated with the indicated low doses of rucaparib and olaparib. (C) Colony formation assays (CFAs) in sensitive parental PEO1 cells and (D) veliparib resistant PEO1/ABTr#3 cells were performed with the indicated concentrations of PG545 alone or in combination with rucaparib. Heat map from the results of CFAs showed very strong synergy as indicated by the combination index (CI) values for (C) PEO1 and (D) PEO1/ABTr#3 cells.

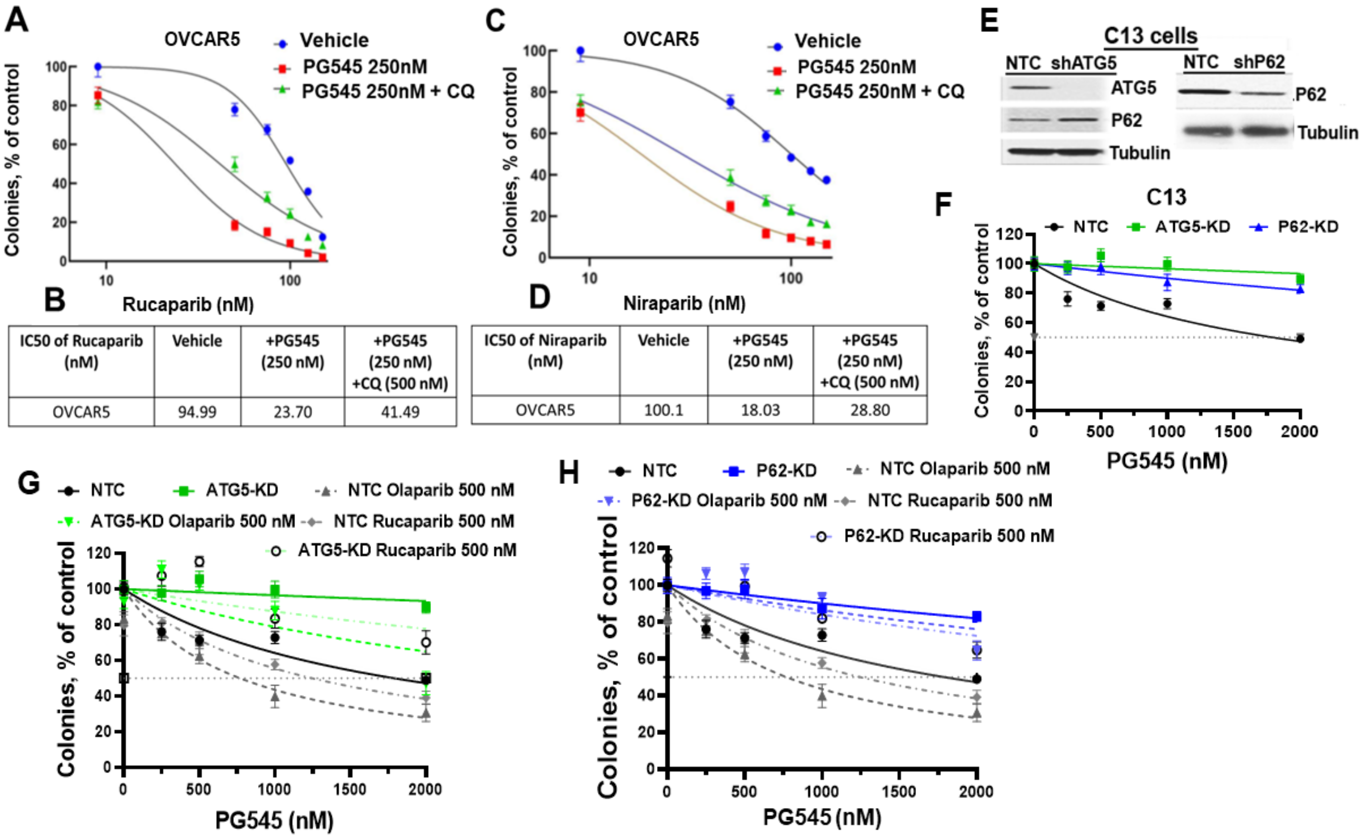

IC50 of Rucaparib (nM)	Vehicle	+PG545 (250 nM)	+PG545 (250 nM) +CQ (500 nM)
OVCAR5	94.99	23.70	41.49

IC50 of Niraparib (nM)	Vehicle	+PG545 (250 nM)	+PG545 (250 nM) +CQ (500 nM)
OVCAR5	100.1	18.03	28.80

Supplementary S2: PG545-induced autophagy sensitizes cells to PARPi-induced cytotoxicity. (A, C) CFAs were performed using OVCAR5 cells treated with the indicated concentrations of PG545 +/- rucaparib or niraparib in the absence or presence of chloroquine (CQ). (B, D) IC_{50} value was calculated and represented as described in the METHODS. (E) Western blot analysis to confirm downregulation of ATG5 and P62 in the knock down C13 cells compared to non-targeted controls. (F) CFAs were performed using NTC control vs ATG5/P62 C13 KD cells treated with the indicated concentrations of PG545. (G) CFAs were performed using NTC control vs ATG5/P62 C13 KD cells treated with the indicated concentrations of PG545 +/- rucaparib/olaparib and (H) PG545 +/- rucaparib/olaparib.

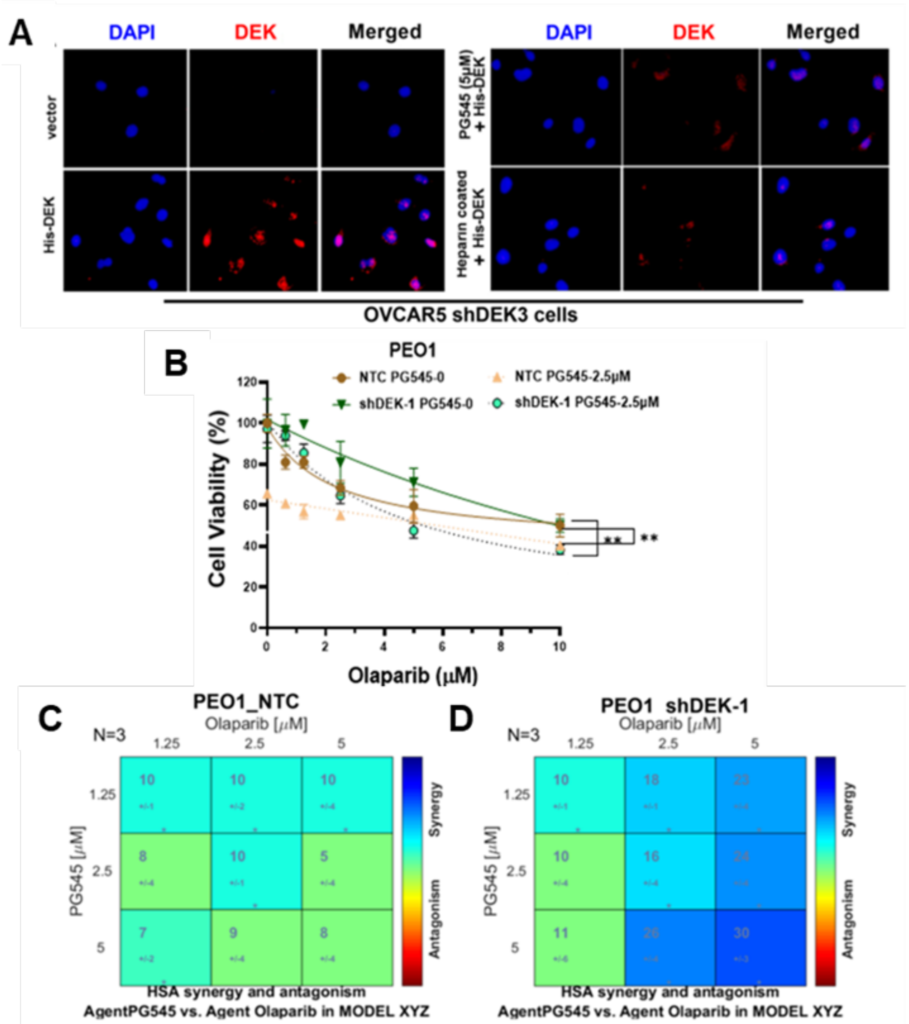

E

	NTC	shDEK-1
PG545 (μM)	Olaparib CI value	
1.25	0.595	0.512
2.5	0.753	0.505
5	0.761	0.344

Supplementary S3: PG545 prevents internalization of DEK protein and DEK knockdown (KD) cells are more sensitive to PG545+olaparib treatment compared to cells transfected with a nontargeting construct (NTC). (A) IF images of OVCAR5 shDEK KD cells transfected with empty vector or His$_6$-tagged DEK in presence of 5μM PG545 or heparin as indicated. Samples were stained for DEK (red). DAPI was used to stain nuclei (blue). (B) Cell viability assay in NTC and DEK KD cells upon treatment with PG545 alone or in combination with

olaparib. (C-D) CFAs using PEO1 NTC (C) or (D) PEO1 DEK KD cells treated with the indicated concentrations of PG545 alone or in combination with olaparib. Response to the combination was analyzed by the HSA synergy and antagonism matrix model using the Combenefit software. Panels show the matrix format of synergy levels calculated according to the HSA synergy and antagonism model from the dual-drug experimental dose response in comparison to the reference dose response surfaces. (E) CI values for the indicated concentrations.

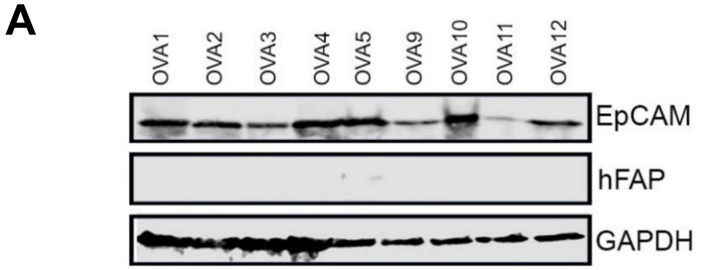

Supplementary S4: Enrichment of epithelial cells from the patient ascites samples.

(A) Western blot analysis of expression of human epithelial specific marker (EpCAM) and human fibroblast marker (FAP) for characterization of epithelial nature of the ascites samples. GAPDH is used as endogenous control.

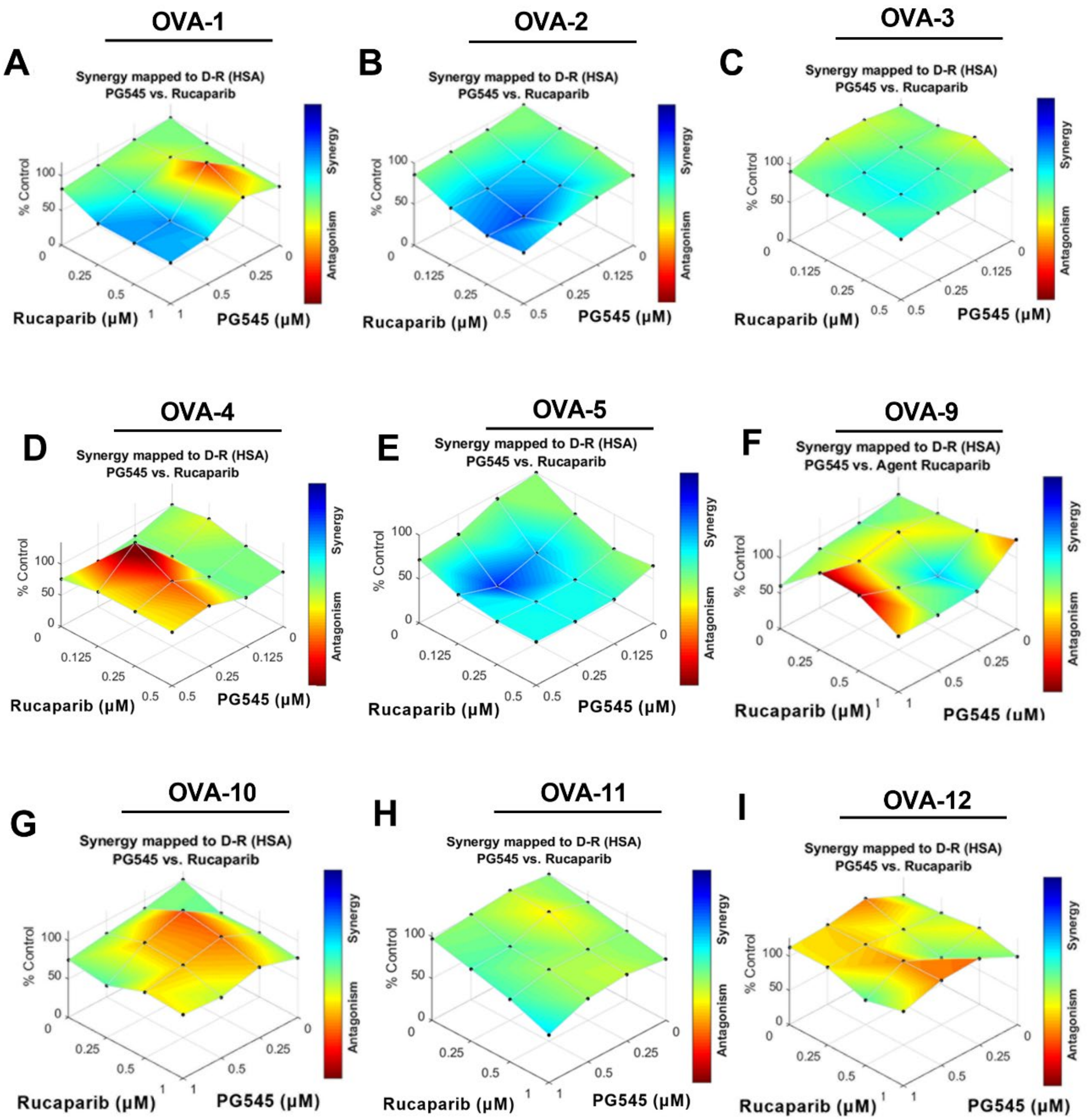

Supplementary S5: Effects of PG545 and rucaparib alone and in combination on patient-derived ascites cells in 3D cultures. Dual drug response assay of PG545 and rucaparib (number of biologic replicates N=3) analyzed by the HSA synergy and antagonism matrix model using the combenefit software. Panels A-I show the combenefit-mapped surface output for the drug combinations involving PG545 and rucaparib in the above-indicated ascites samples, respectively.

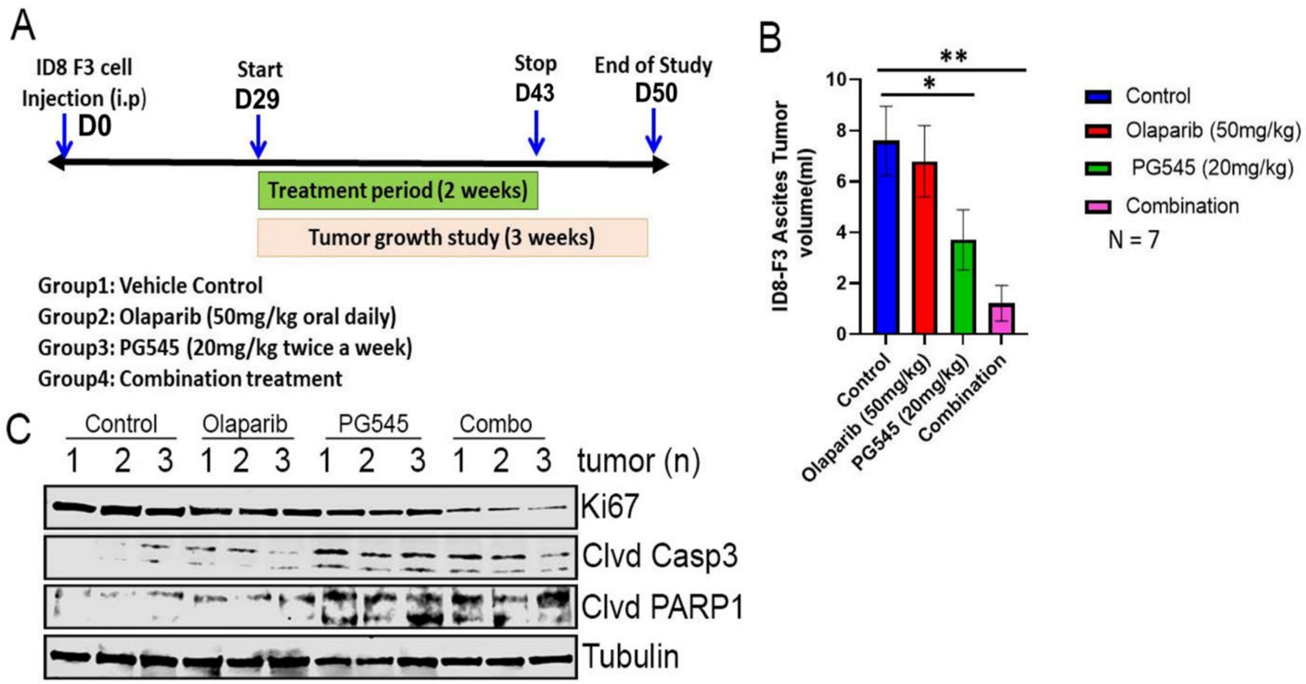

Supplementary S6: Rucaparib and PG545 exhibit synergistic antitumor effects in a syngeneic OC model. (A) Schematic representation of the *in vivo* study using ID8F3 OC cells. (B) Graphical presentation of the ascites volume in the control vs treatment cohorts (N=7, **p<0.01, *p<0.05). (C) Western blot analysis of Ki67, cleaved PARP and cleaved caspase3 from the ascites samples of the control and treated groups. Tubulin was used as a loading control.
